# Supplementary material for: Existence of edge modes in periodic microstrip transmission line
Source: Sci Rep. 2024 Jul 16;14:16477. doi: 10.1038/s41598-024-67610-9 (PMC11252309; doi:10.1038/s41598-024-67610-9)
Supplement: Supplementary file 1 — Supplementary Information. [file 41598_2024_67610_MOESM1_ESM.pdf]

Supplementary Information:

Existence of edge modes in periodic microstrip transmission line

Aleksey Girich[1], Liubov Ivzhenko[1,2*], Ganna Kharchenko[1], Sergey Polevoy[1], Sergey Tarapov[1,3,4], Maciej Krawczyk[2], and Jarosław W. Kłos[2]

[1]O. Ya. Usikov Institute for Radiophysics and Electronics NAS of Ukraine, Kharkiv,Ukraine
[2]ISQI, Faculty of Physics, Adam Mickiewicz University, Poznań, Poland
[3]Gebze Technical University, Kocaeli, Turkey
[4]V.N. Karazin Kharkiv National University, Kharkiv, Ukraine
[*]ivzhenko@amu.edu.pl

ABSTRACT

The microstrip of modulated width is a realization of a one-dimensional photonic crystal operating in the microwave regime. Like any photonic crystal, the periodic microstrip is characterised by the presence of frequency bands and band gaps that enable and prohibit wave propagation, respectively. The frequency bands for the microstrip of the symmetric unit cell can be distinguished by 0 or π Zak phase. The sum of these topological parameters for all bands below a given frequency gap determines the value of the surface impedance and whether or not edge modes are present at the end of the microstrip. We demonstrate that edge modes are absent in a finite microstrip terminated at both ends in the centres of unit cells, but they can be induced by adding the defected cells. Edge modes present at both ends of the microstrip enable microwave tunneling with high transitivity in the frequency gap with or without a change in phase. This has been demonstrated experimentally and developed in detail using numerical simulations and model calculations. The investigated system, with a doublet of edge modes in the frequency gap, can be considered as a narrow passband filter of high selectivity.

S1. Impedance and effective permittivity for uniform microstrip

The effective permittivities and impedances for wider (ε_A, Z_A) and narrower (ε_B, Z_B) segments of the microstrip (see Fig.1 in the manuscript) were calculated using the approximate formulas[1]:

$$\varepsilon_{A,B} = \frac{\varepsilon+1}{2} + \frac{\varepsilon-1}{2\sqrt{1+12\,r_{A,B}}}, \tag{S1}$$

and

$$Z_{A,B} = \frac{120\pi}{\sqrt{\varepsilon_{A,B}}\left(r_{A,B}+1.393+\frac{2}{3}\left(r_{A,B}+1.444\right)\right)}, \tag{S2}$$

where $r_{A,B} = w_{A,B}/u$. In periodic microstrip the effective permittivity and impedance can be considered as functions of the $z-$coordinate: $\varepsilon(z)$, $Z(z)$, taking two alternative values: ε_A, ε_B and Z_A, Z_B as z is increasing along the microstrip.

S2. Bloch impedance

In the general case, the Bloch impedance can also be expressed in terms of the logarithmic derivatives of the Bloch function, namely $\rho(k,z)$ of $E_k(z)$ and $H_k(z)$, expressed as $\partial_z \ln(E_k(z))$ or $\partial_z \ln(H_k(z))$. This can be demonstrated by utilizing the following relations resulting from Maxwell's equations: $\partial_z E_k(z) = i\omega\mu_0 H_k(z)$, $\partial_z H_k(z) = i\omega\varepsilon\varepsilon_0 E_k(z)$:

$$Z_B(z) = -i\frac{\partial_z \ln(H_k(z))}{\mu_0\omega} = i\frac{\varepsilon_0\varepsilon(z)\omega}{\partial_z \ln(E_k(z))}. \tag{S3}$$

In the frequency gaps, the logarithmic derivative of the Bloch function is a real-valued function of the frequency and reaches the zeros and poles at the edges of the gaps (depending on the symmetry of the Bloch function)[2]. These properties justify the characteristics of the Bloch impedance in the frequency gaps as $Z_B = i\xi$.

It is also known that the logarithmic derivative of the Bloch function $\rho(k,z)$ has the following property[2–4]:

$$\rho(k,-z) = -\rho(-k,z)^*, \tag{S4}$$

where the asterisk '*' denotes a complex conjugation. This property means that in a frequency gap where ρ is real and $k = n\pi/d + ik_I$, changing the side of the interface/surface ($x \leftrightarrow -x$) and the direction of the decay for the interface/surface mode ($k_I \leftrightarrow -k_I$) results in the fillip of the logarithmic derivative and the imaginary part of the Bloch impedance ($\xi \leftrightarrow -\xi$).

The logarithmic derivative taken at the boundary of the centrosymmetric unit cell $x = x_0$ can change its sign if we select the centrosymmetric cell alternatively (i.e. for $z_0 \to z_0 + d/2$). The change of sign is observed in every second gap, i.e. for the gaps $n = 1,3,5,\ldots$, which are located at the edge of the 1st Brillouin zone:

$$\rho(\pi/d + ik_I, z_0) = -\rho(\pi/d + ik_I, z_0 + d/2). \tag{S5}$$

Changing $z_0 \to z_0 + d/2$ results in reversing the sign of the imaginary part of the Bloch impedance ($\xi \leftrightarrow -\xi$) in every second gap: $n = 1,3,5,\ldots$.

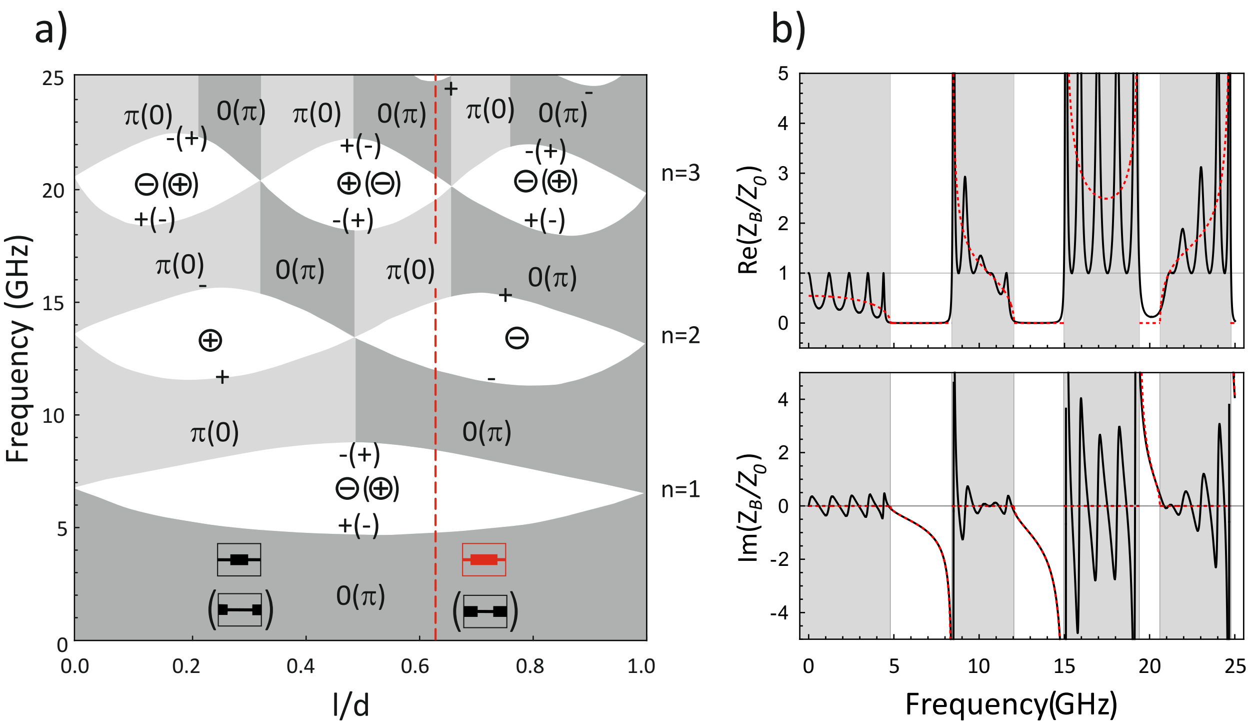

Figure S1. (a) Band structure of an infinite periodic microstrip calculated using a 1D model of an equivalent photonic crystal as a fraction of the filling fraction: ratio of the length of the wider segment l to the lattice constant d. Grey (white) areas mark the frequency bands (gaps). The symbols $+$ and $-$ denote the symmetric and antisymmetric profiles of the Bloch function at the edges of the bands. The values 0 and π refer to the Zak phase in the given band, while the symbols $\oplus$ and $\ominus$ indicate the sign of the logarithmic derivative in the frequency gap (at the left edge). The symbols and values given with or without brackets refer to two possible choices of symmetric unit cells. The experimental value of the ratio $l/d = 0.625$ is marked by the vertical dashed line. (b) The real (upper plot) and imaginary (lower plot) parts of the Bloch impedance in the infinite system (red dashed line) and its approximation in the finite microstrip composed of $N = 5$ cells (black line).

To illustrate the frequency dependence of the Bloch impedance, we can consider a 1D model[5] of a photonic crystal equivalent to a periodic stepped-impedance microstrip[6]. The impedances and effective permittivities of homogeneous microstrips, corresponding to the widths of the two microstrip sections, have been assigned to the two different layers of the photonic crystal. This 1D model is more accurate than the lumped-element model, but it still exhibits the deficiencies that prevent the correct determination of edge modes and antiresonance in the system we considered.

The Bloch impedance can be calculated directly for the infinite system, if we know the transfer matrix $\mathbf{T}$ for the unit cell and apply the Bloch theorem[1,4]. For a centro-symmetric unit cell ($T_{11} = T_{22}$) the Bloch impedance can be written in the following form[1]:

$$Z_B = \frac{T_{12}}{\sqrt{T_{11}^2 - 1}}. \tag{S6}$$

For the finite structure, we can calculate the impedance on the left end of the system by using the very general formula:

$$Z_{\text{in}} = Z_0 \frac{1+S_{11}}{1-S_{11}} \underset{\text{in the gaps}}{\approx} Z_B, \tag{S7}$$

where S_{11} is the reflection coefficient calculated as an element of the scattering matrix $\mathbf{S}$ of the whole system. The value of Z_{in} approximates the Bloch impedance Z_B very well in the frequency gaps. In the frequency bands Z_{in} oscillate with the change in frequency. The oscillations are centred around the values of Z_B and their number increases with the number of unit cells in the system.

S3. Lumped element model for periodic microstrip

The full electromagnetic simulations can be supplemented by semi-analytical calculations, which can give deeper insight into physical mechanisms or just provides a description that is more intuitive or familiar to a wider audience. The microstrips of stepper impedance are often described using the equivalent model of 1D layered photonic structure (see Supplementary Information S2). However, using this 1D approach does not correctly reproduce the frequency of edge modes, nor the presence of anti-resonance. The periodic microstrip is a transmission line that can be roughly approximated by a lumped-element model as a ladder network (see Fig. S2(a)), which is commonly used in the engineering community. Each unit cell of the structures can be described as a phase shifter with shunt capacitance. The phase shift depends on the length of the unit cell d and the wave vector $k_0 = 2\pi f/(c/\sqrt{\varepsilon_{\text{eff}}})$ in the medium characterized by the dielectric constant ε_{eff}, where the symbol c denotes the speed of light. The shunt capacitance C is related to the capacitance of a single section of microstrip deposited on a dielectric substrate with a ground plane at the bottom. The formal relationship between output and input voltages (V_j) and currents (I_j) for each segment (j^{th}) of the network is described by the so-called ABCD-matrix[1], with frequency-dependent elements.

The formal relationship between output and input voltages (V_j) and currents (I_j) for each segment (j^{th}) of the lumped-element model network is described by the ABCD-matrix (transfer matrix) $\mathbf{T}$:

$$\begin{pmatrix} V_j \\ I_j \end{pmatrix} = \mathbf{T} \begin{pmatrix} V_{j+1} \\ I_{j+1} \end{pmatrix}. \tag{S8}$$

For our system, the matrix has a form:

$$\mathbf{T} = \begin{pmatrix} \cos\Omega - p\Omega\sin(\Omega) & i\left(p\left(\cos\Omega - 1\right) + \sin\Omega\right) \\ i\left(p\left(\cos\Omega + 1\right) + \sin\Omega\right) & \cos\Omega - p\Omega\sin(\Omega) \end{pmatrix}, \tag{S9}$$

where $\Omega = k_0 d$ is the dimensionless angular frequency. The symbol p is defined as $p = q(c/\sqrt{\varepsilon_{\text{eff}}}(1/(2d))$, where $q = Z_0 C$.

In an infinite network, the voltages and currents in successive cells are related by the wavenumber k:

$$V_j = e^{ik_z d} V_{j+1}, \quad I_j = e^{ik_z d} I_{j+1}. \tag{S10}$$

The lumped-element model contains two parameters: ε_{eff} and p, which are related to the geometric and material parameters of the real MSTL. We have tuned the values of ε_{eff} and p to obtain positions of the stop bands that are the same as those found from the experiment and numerical simulations. Eq. S10 allowed us to find the dispersion relation $f(k_z)$ for infinite network, The resulting transmission for the finite structure can be calculated as a product of the $\mathbf{T}$ matrices (S9) for successive networks. This matrix can then be converted into the corresponding scattering matrix $\mathbf{S}$, which allows to obtain the transmission spectrum by taking its S_{21} element[1].

To relate the lumped-element model to the full electromagnetic model used in numerical calculations, we adjusted the values of two parameters C and ε_{eff}. The accuracy of the lumped-element model is limited, and we could not properly reproduce the spectrum in a wide frequency range, covering many bands and gaps. In this study, we matched the position and width of the second frequency gaps of the infinite ladder network and periodic microstrip for the values $C = 0.48$ pF and $\varepsilon_{\text{eff}} = 1.6$. The geometric changes of the edge cells in finite systems are introduced by varying the parameters: C_0 and d_0. The length of the edge cells d_0 is clearly defined ($d_0 = 11.5$ mm for the experimental realization). We have estimated the value of C_0

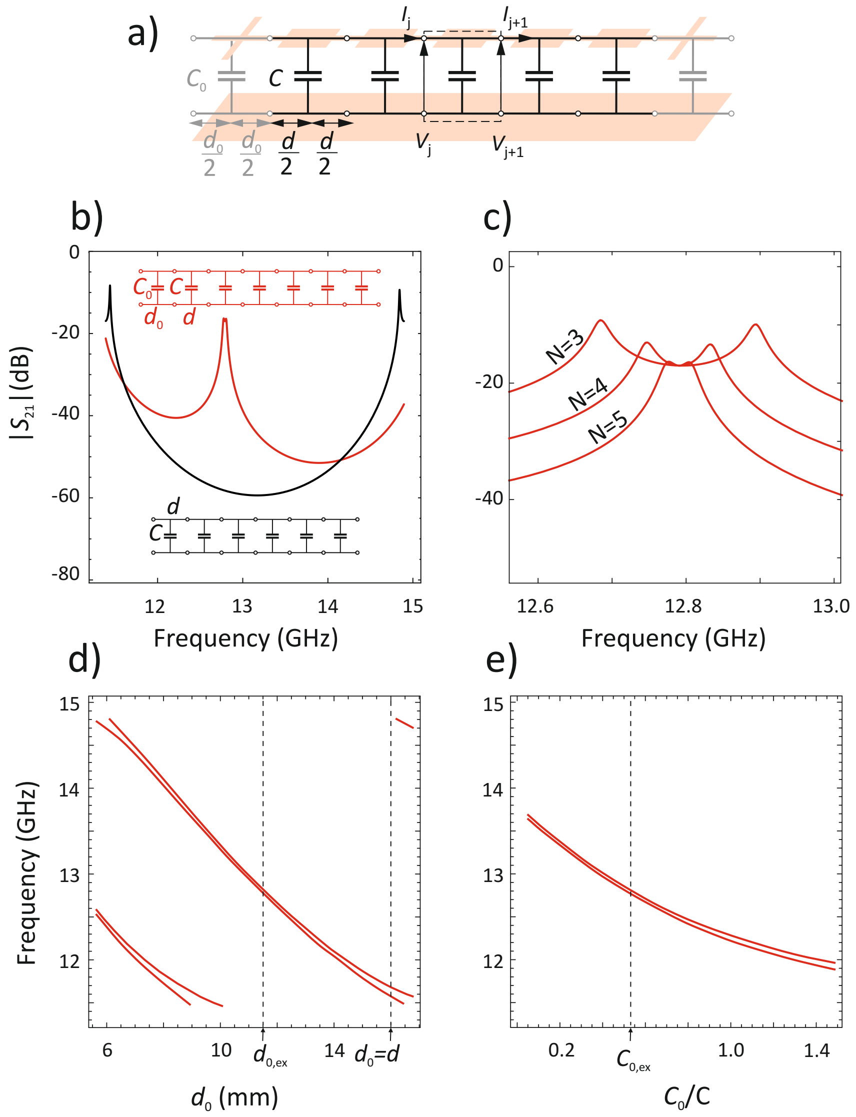

Figure S2. (a) The lumped element model of the finite periodic microstrip, where the bulk and edge cells are represented by the symmetric two-port networks. Each network is composed of two phase shifters (resulting from the acquisition of the phase at the distance $d/2$ and $d_0/2$, respectively – see Chapter 8.1 in Ref. 1) and shunt susceptances (resulting from the presence of the capacitance C and C_0, respectively, in each cell of the system). Results for the lumped-element model. (b) The transmission spectrum within the second frequency gap for a ladder network is composed of five identical cells (black line) and extended by two cells with different parameters: $d_0 \neq d$ and $C_0 \neq C$ (red line). (c) The magnified peak for the doublet of edge modes becomes split when the number of bulk cells N is reduced. (d,e) Tuning the edge mode frequency. We studied the effect of the length of the edge cell d_0 (d) and the shunt capacitance C_0 (e). In (d) we fixed the shunt capacitance $C_0 = 0.53C$, and in (e) – the length $d_0 = 0.73d$ for the edge cells.

assuming that the ratio of the area of the edge cell to the area of the bulk cell: S_0/S can be related to the corresponding ratio of capacitances: $S_0/S \approx C_0/C$. By calculating the transmission for the ladder network, we found that the transmission peak is located inside the gap, close to the experimentally determined frequency – see Fig. S2(b). Careful inspection allows us to note that the peak is doubled – see Fig. S2(c).

In Fig. S2(d,e) we show the dependence of the edge mode frequencies on d_0 and C_0 in the second frequency gap for the lumped-element model. By changing the length of the edge cell d_0 from 6 to 19 mm, we shift the edge mode doublet from the top to the bottom of the frequency gap – Fig. S2(d). The doublet increases the splitting near the edges of the frequency gap.

This is due to the imaginary part of the wavenumber, which has a maximum value at the center of the gap and decreases as one moves toward the edges. Close to the frequency band, the surface modes are less localized and the excitations at both edges of the microstip are strongly coupled. The lumped-element model predicts that for small values of d_0 (less than 10 mm) an additional doublet of surface modes appears at the bottom of the frequency gap. However, such states were not found using numerical simulations (Fig. 3(c) in the manuscript) in the investigated range of d_0. We assume that this is an artifact introduced by the lumped-element model. The frequency of edge modes can also be tuned by the shunt capacitance C_0 in the lumped element model (see Fig. S2(e)). The increase of the capacitance for the edge cells of the network can be related to the widening of the central segment in the edge cells of the microstrip. In both models, the frequency of the edge modes is reduced with the increase of C_0 (w_0). The central sections of the edge cells act as stubs, which are typically distributed elements. The edge mode frequencies are below the anti-resonance frequency of the stubs. In this frequency range, transmission through the stub feed decreases as the antiresonance frequency is approached. Moreover, as the stub length $w_0/2$ grows, the impedance increase is greater because the anti-resonance frequency is reduced. A shunt capacitor has a similar characteristic: as the frequency increases, the shunt impedance increases (the signal is shorted to ground), and as w_0 is extended, the capacitance C_0 increases, and the variation of impedance with frequency is greater. For these reasons, the shunt capacitor qualitatively reproduces the operation of the stub in the narrow frequency range corresponding to the second frequency gap.

The frequency difference between the two edge modes decreases as the number of cells increases – see Fig. S2(c). This can be understood by noting that for a larger number of cells $N > 5$, the amplitudes of both modes practically decay to zero inside the periodic microstrip. As a result, the microwave fields at both edges do not affect each other. For such systems, it is very difficult to choose the frequency that allows microwave-field tunneling with 0 or π phase shift between the input and output port (see Fig. 4 in the manuscript).

S4. Anti-resonances related to the presence of edge cells

Figure S3 shows the simulated transmission spectra $|S_{21}|(f)$ through the single edge cell connected to the ports of the impedance $Z_0 = 50\ \Omega$, for different widths of the central section w_0, which play a role of the two open stubs attached at the same location on both sides of the feed line. In the transmission spectra, we can see sharp anti-resonances for the frequencies 15 GHz and 21 GHz, corresponding to the stub lengths $w_0 = 9$ mm and 6 mm respectively. It is easy to prove that these frequencies give rise to the $\lambda/4$ anti-resonances: $\frac{w_0}{2}k_0 = \pi/2$. From this condition, the anti-resonance frequency can be estimated. This can be done by relating the frequency to the wave vector $k_0 = 2\pi f/(c/\sqrt{\varepsilon_{\text{stub}}})$ for a standing wave in the stub, where the effective permittivity in stub $\varepsilon_{\text{stub}}$ is calculated from (S1). The estimated values of the anti-resonances are 14.5 GHz (for $w_0 = 9$ mm) and 19.4 GHz (for $w_0 = 6$ mm), which correspond to the numerical results for a single edge cell. These results are also consistent with the anti-resonances observed for the microstrip (Fig. 5(a) in the manuscript).

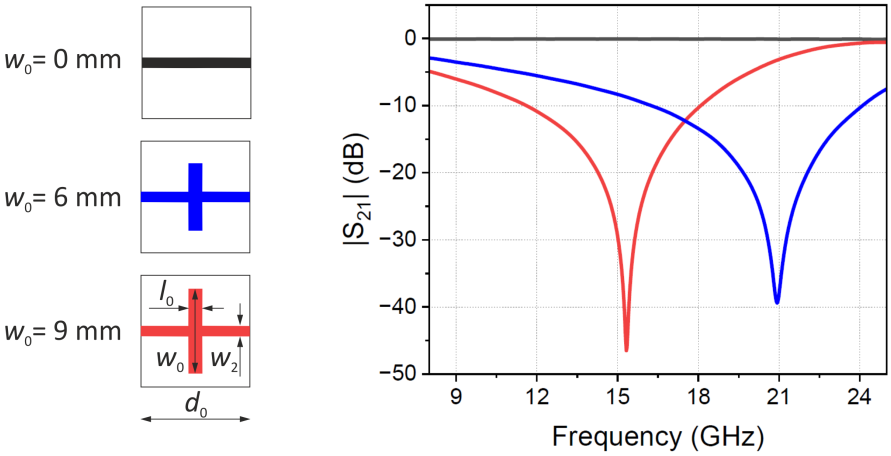

Figure S3. The numerically calculated transmission spectra through the edge cell, whose central part forms a double stub. The frequency of the anti-resonance is increased as the length of the stub (width of the central section w_0) is increased. The values $d_0 = 11.5$ mm, $l_0 = 1.5$ mm, $w_2 = 1.12$ mm are fixed and correspond to the experimental realization, where the width of the central section is equal to $w_0 = 9$ mm.

S5. Group delay for edge modes

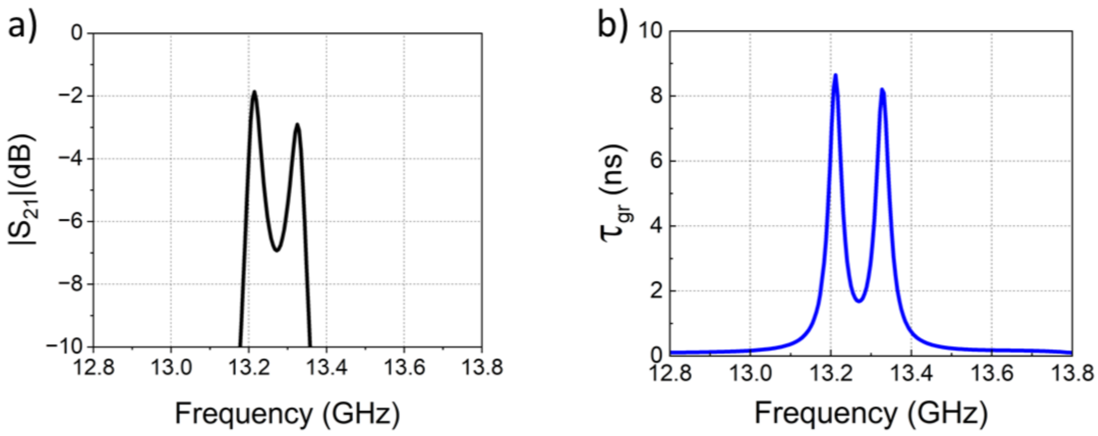

Figure S4. Results of numerical simulations showing (a) the trasmission spectrum in the second frequency gap with a doublet of edge modes and (b) the group delay for this doublet.

The edge modes are characterised by a large group delay τ_{gr} which reaches a peak values of about 8 ns – see, Fig. S4. The dependence τ_{gr} on frequency is non-monotonic and is characterised by a deep minimum for frequencies between doublet peaks, i.e. in the region where the transmission is still quite high. The rising and falling edges of the $\tau_{gr}(f)$ dependence can be used to map signals from the frequency domain to the time domain.

References

1. Pozar, D. M. *Microwave Engineering* (John Wiley and Sons, 2011).
2. Zak, J. Symmetry criterion for surface states in solids. *Phys. Rev. B* **32**, 2218, DOI: 10.1103/PhysRevB.32.2218 (1985).
3. Mieszczak, S. & Kłos, J. W. Interface modes in planar one-dimensional magnonic crystals. *Sci. Rep.* **12**, 11335, DOI: 10.1038/s41598-022-15328-x (2022).
4. Tsukerman, I. & Markel, V. A. Topological features of Bloch impedance. *Eur. Lett.* **144**, 16002, DOI: 10.1209/0295-5075/acfe93 (2023).
5. Xiao, M., Zhang, Z. Q. & Chan, C. T. Surface impedance and bulk band geometric phases in one-dimensional systems. *Phys. Rev. X* **4**, 021017, DOI: 10.1103/PhysRevLett.48.359 (2014).
6. Nakata, Y., Ito, Y., Nakamura, Y. & Shindou, R. Topological boundary modes from translational deformations. *Phys. Rev. Lett.* **124**, 073901, DOI: 10.1103/PhysRevLett.124.073901 (2020).
